# A crystallographically isolated dimeric hydrolyzed chloro­phosphazene dianion

**DOI:** 10.1107/S1600536808042116

**Published:** 2008-12-20

**Authors:** Matthew J. Panzner, Wiley J. Youngs, Claire A. Tessier

**Affiliations:** a The University of Akron, Chemistry Department, Akron, OH 44325-3601, USA

## Abstract

Single crystals of the title compound bis[bis­(1-ethyl-3-methyl-imidazol-2-yl­idene)silver(I)] 1,5,5,7,11,11-hexa­chloro-2,8-di­oxa-4,6,10,12,13,14-hexa­aza-1λ^5^,3,5λ^5^,7λ^5^,9,11λ^5^-hexa­phospha­tricyclo­[7.3.1.1^3,7^]tetra­deca-1(13),4,7(14),10-tetra­ene-6,12-diide 3,9-dioxide, [Ag(C_6_H_10_N_2_)_2_](Cl_6_N_6_O_4_P_6_)_0.5_, were isolated from the reaction of the silver *N*-heteocyclic carbene complex [Ag(C_6_H_10_N_2_)_2_]Cl and hexa­chloro­cyclo­triphos­phazene [NPCl_2_]_3 _in the presence of water. The asymmetric unit contains one silver carbene cation with the carbene ligands bound to the Ag(I) in an almost linear arrangement and one half of a hydrolyzed phosphazene dianion. The second cation and additional half of the anion are generated by an inversion center.

## Related literature

For background on phosphazene hydrolysis products, see: Allcock (2003 ▶); Allcock *et al.* (1975 ▶); Gabler & Haw (1990 ▶); Murray *et al.* (1994 ▶); van de Grampel (1992 ▶). For related structures, see: Bartlett *et al.* (2006 ▶); Brandt *et al.* (1991 ▶); Bullen (1971 ▶); Meetsma *et al.* (1990 ▶).
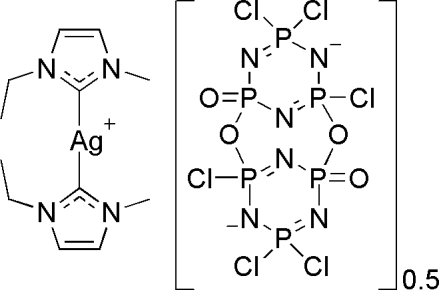


## Experimental

### 

#### Crystal data

[Ag(C_6_H_10_N_2_)_2_](Cl_6_N_6_O_4_P_6_)_0.5_
*M* *_r_* = 601.48
Triclinic, 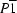

*a* = 9.3224 (15) Å
*b* = 10.6190 (18) Å
*c* = 12.257 (2) Å
α = 78.916 (3)°
β = 71.558 (3)°
γ = 76.107 (3)°
*V* = 1108.4 (3) Å^3^
*Z* = 2
Mo *K*α radiation
μ = 1.51 mm^−1^
*T* = 100 (2) K
0.30 × 0.08 × 0.04 mm

#### Data collection

Bruker SMART CCD area-detector diffractometer
Absorption correction: multi-scan (*SADABS*; Bruker, 2001 ▶) *T*_min_ = 0.660, *T*_max_ = 0.942
8912 measured reflections
4459 independent reflections
3484 reflections with *I* > \2s(*I*)
*R*_int_ = 0.031

#### Refinement

*R*[*F* ^2^ > 2σ(*F* ^2^)] = 0.047
*wR*(*F* ^2^) = 0.123
*S* = 1.04
4459 reflections
257 parameters
H-atom parameters constrained
Δρ_max_ = 2.25 e Å^−3^
Δρ_min_ = −1.02 e Å^−3^

### 

Data collection: *SMART* (Bruker, 2007 ▶); cell refinement: *SAINT* (Bruker, 2007 ▶); data reduction: *SAINT*; program(s) used to solve structure: *SHELXS97* (Sheldrick, 2008 ▶); program(s) used to refine structure: *SHELXL97* (Sheldrick, 2008 ▶); molecular graphics: *SHELXTL* (Sheldrick, 2008 ▶); software used to prepare material for publication: *SHELXTL*.

## Supplementary Material

Crystal structure: contains datablocks I, global. DOI: 10.1107/S1600536808042116/br2087sup1.cif

Structure factors: contains datablocks I. DOI: 10.1107/S1600536808042116/br2087Isup2.hkl

Additional supplementary materials:  crystallographic information; 3D view; checkCIF report

## Figures and Tables

**Table:** 

Ag—C1	2.065 (5)
Ag—C7	2.070 (5)
Cl1—P2	2.0118 (16)
Cl2—P2	2.0171 (17)
Cl3—P3	2.0217 (16)
P1—O1	1.468 (3)
P1—N5	1.610 (4)
P1—N6	1.615 (4)
P1—O2	1.657 (3)
P2—N6	1.555 (4)
P2—N7	1.578 (4)
P3—N5^i^	1.554 (4)
P3—O2	1.578 (3)
P3—N7^i^	1.594 (4)

**Table:** 

C1—Ag—C7	178.72 (19)

Symmetry code: (i) 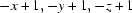.

**Fig. 1.**
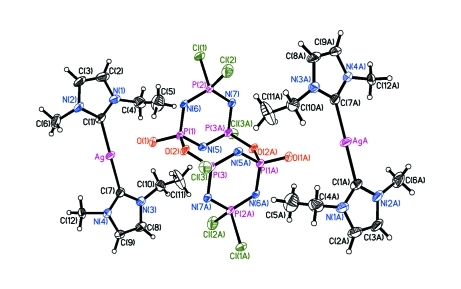
Structure of the title compound, first crystallographically characterized dimeric oxygen-bridged chlorophosphazene dianion. A numbering scheme of the non-H atoms is shown and thermal ellipsoids shown at 50% probability.

## supplementary crystallographic information

### Comment

Hexachlorocyclotriphosphazene [NPCl_2_]_3_ is used as a starting material for the synthesis of poly(dichlorophosphazene). This conversion can be achived by a ring opening melt polymerization process at high temperature, generally greater than 473 °K. It has long been contested that trace amounts of water not only accelerate the rate of polymerization but are necessray to generate active species needed to simply promote polymer formation (Allcock, 2003; Allcock *et al.*, 1975). Isolation of hydrolyzed species gives insight into the still unclear role that water plays in this polymerization reaction (Gabler *et al.*, 1990). As part of our broader studies on the irreproducibility in the synthesis of poly(dichlorophosphazene) we report the first crystal structure of a dimeric hydrolyzed chlorophosphazene dianion.

The asymmetric unit consists of one silver N-heteocyclic carbene cation [AgC_12_H_20_N_4_]^+^ and half of a hydrolyzed phosphazene dianion [Cl_6_N_6_O_4_P_6_]^2-^. The second cation and other half of the dianion are generated by a crystallographic inversion center at *x*,*y*,*z* (1/2,1/2,1/2) located between one of the bridging oxygen atoms of the dimeric dianion and its symmetry generated equivalent. Each of the Ag(I) atoms is bound to two identical N-heterocyclic carbene ligands in a linear fashion. Two partially hydrolyzed phosphazene rings are joined by bridging oxygen atoms from a phosphorus atom of one ring to the other to form the dimeric dianion. The P—O—P bonds of the bridging oxygen atoms are inequivalent . The P—N bond distances of the individual rings deviate from the reported values of hexachlorocyclotriphosphazene, all six being virtually equivalent (Bartlett *et al.*, 2006; Bullen, 1971). Two of the P—N bonds on each of the rings show significant double bond character while the remaining P—N bonds are lengthened to give more single bond character.

### Experimental

The title compound, bis[bis(1-ethyl-3-methylimidazol-2-ylidene)silver(I)] 1,5,5,7,11,11-hexachloro-2,8-dioxa-4,6,10,12,13,14-hexaaza-1λ^5^,3,5λ^5^,7λ^5^,9,11λ^5^-hexaphosphatricyclo[7.3.1.1^3,7^]tetradeca-1(13),4,7(14),10-tetraene-6,12-diide 3,9-dioxide, [Ag(C_6_H_10_N_2_)_2_](Cl_6_N_6_O_4_P_6_)_0.5_, was isolated from the reaction of silver N-heteocyclic carbene complex [AgC_12_H_20_N_4_]^+^.Cl^-^ (generated *in situ*) and hexachlorocyclotriphosphazene, [NPCl_2_]_3_. A small amount of crystals were obtained after removing volatiles from the reaction.

### Refinement

Hydrogen atoms were calculated and palced in geometrically idealized positions with C—H distances of 0.95 Å (aromatic), 0.99 Å (methylene), and 0.98 Å (methyl). H atoms were constrained to ride on the parent carbon atom with *U*_iso_(H) = 1.2 *U*_eq_(C) for aromatic and methylene and *U*_iso_(H) = 1.5 *U*_eq_(C) for methyl protons.

### Crystal data

**Table:**

[Ag(C_6_H_10_N_2_)_2_](Cl_6_N_6_O_4_P_6_)_0.5_	*Z* = 2
*M**_r_* = 601.48	*F*(000) = 600
Triclinic, *P*1	*D*_x_ = 1.802 Mg m^−^^3^
Hall symbol: -P 1	Mo *K*α radiation, λ = 0.71073 Å
*a* = 9.3224 (15) Å	Cell parameters from 2618 reflections
*b* = 10.6190 (18) Å	θ = 2.5–26.8°
*c* = 12.257 (2) Å	µ = 1.51 mm^−^^1^
α = 78.916 (3)°	*T* = 100 K
β = 71.558 (3)°	Plate, colourless
γ = 76.107 (3)°	0.30 × 0.08 × 0.04 mm
*V* = 1108.4 (3) Å^3^	

### Data collection

**Table:**

Bruker SMART CCD area-detector diffractometer	4459 independent reflections
Radiation source: fine-focus sealed tube	3484 reflections with *I* > \2s(*I*)
graphite	*R*_int_ = 0.031
φ and ω scans	θ_max_ = 26.3°, θ_min_ = 1.8°
Absorption correction: multi-scan (*SADABS*; Bruker, 2001)	*h* = −11→11
*T*_min_ = 0.660, *T*_max_ = 0.942	*k* = −13→13
8912 measured reflections	*l* = −15→15

### Refinement

**Table:**

Refinement on *F*^2^	Primary atom site location: structure-invariant direct methods
Least-squares matrix: full	Secondary atom site location: difference Fourier map
*R*[*F*^2^ > 2σ(*F*^2^)] = 0.047	Hydrogen site location: inferred from neighbouring sites
*wR*(*F*^2^) = 0.123	H-atom parameters constrained
*S* = 1.04	*w* = 1/[σ^2^(*F*_o_^2^) + (0.0737*P*)^2^] where *P* = (*F*_o_^2^ + 2*F*_c_^2^)/3
4459 reflections	(Δ/σ)_max_ < 0.001
257 parameters	Δρ_max_ = 2.25 e Å^−^^3^
0 restraints	Δρ_min_ = −1.02 e Å^−^^3^

### Special details

**Table:**

Geometry. All e.s.d.'s (except the e.s.d. in the dihedral angle between two l.s. planes) are estimated using the full covariance matrix. The cell e.s.d.'s are taken into account individually in the estimation of e.s.d.'s in distances, angles and torsion angles; correlations between e.s.d.'s in cell parameters are only used when they are defined by crystal symmetry. An approximate (isotropic) treatment of cell e.s.d.'s is used for estimating e.s.d.'s involving l.s. planes.
Refinement. Refinement of *F*^2^ against ALL reflections. The weighted *R*-factor *wR* and goodness of fit *S* are based on *F*^2^, conventional *R*-factors *R* are based on *F*, with *F* set to zero for negative *F*^2^. The threshold expression of *F*^2^ > σ(*F*^2^) is used only for calculating *R*-factors(gt) *etc*. and is not relevant to the choice of reflections for refinement. *R*-factors based on *F*^2^ are statistically about twice as large as those based on *F*, and *R*- factors based on ALL data will be even larger.

### Fractional atomic coordinates and isotropic or equivalent isotropic displacement parameters (Å^2^)

**Table:**

	*x*	*y*	*z*	*U*_iso_*/*U*_eq_	
Ag	0.92396 (4)	0.82923 (3)	0.02673 (3)	0.02921 (14)	
Cl1	0.20816 (15)	0.96514 (11)	0.50044 (11)	0.0364 (3)	
Cl2	0.06691 (14)	0.72027 (14)	0.58126 (11)	0.0402 (3)	
Cl3	0.32100 (13)	0.28263 (12)	0.39490 (11)	0.0312 (3)	
P1	0.50886 (13)	0.63373 (11)	0.34302 (10)	0.0220 (3)	
P2	0.27569 (13)	0.76965 (11)	0.50900 (10)	0.0218 (3)	
P3	0.46494 (13)	0.36960 (11)	0.43519 (10)	0.0196 (3)	
O1	0.5964 (4)	0.6691 (3)	0.2226 (3)	0.0298 (8)	
O2	0.4577 (3)	0.4948 (3)	0.3425 (3)	0.0243 (7)	
N1	0.6862 (5)	1.0763 (4)	0.0964 (4)	0.0346 (10)	
N2	0.6984 (5)	1.0504 (4)	−0.0734 (4)	0.0330 (10)	
N3	1.1582 (4)	0.6223 (4)	0.1266 (3)	0.0298 (9)	
N4	1.1637 (4)	0.5782 (4)	−0.0374 (3)	0.0277 (9)	
N5	0.6039 (4)	0.5991 (3)	0.4376 (3)	0.0219 (8)	
N6	0.3458 (4)	0.7321 (4)	0.3838 (3)	0.0259 (9)	
N7	0.3694 (4)	0.7221 (3)	0.6012 (3)	0.0207 (8)	
C1	0.7588 (5)	0.9961 (4)	0.0151 (4)	0.0287 (11)	
C2	0.5818 (6)	1.1759 (5)	0.0603 (6)	0.0454 (15)	
H2	0.5168	1.2441	0.1033	0.055*	
C3	0.5876 (6)	1.1600 (5)	−0.0462 (5)	0.0414 (14)	
H3	0.5274	1.2135	−0.0937	0.050*	
C4	0.7138 (7)	1.0544 (6)	0.2095 (5)	0.0455 (14)	
H4A	0.8242	1.0172	0.2013	0.055*	
H4B	0.6891	1.1391	0.2404	0.055*	
C5	0.6220 (9)	0.9672 (7)	0.2893 (6)	0.0638 (19)	
H5A	0.5127	1.0074	0.3028	0.096*	
H5B	0.6483	0.9494	0.3629	0.096*	
H5C	0.6423	0.8850	0.2569	0.096*	
C6	0.7431 (8)	0.9969 (6)	−0.1845 (5)	0.0513 (16)	
H6A	0.8545	0.9887	−0.2195	0.077*	
H6B	0.6889	1.0558	−0.2370	0.077*	
H6C	0.7155	0.9106	−0.1707	0.077*	
C7	1.0921 (5)	0.6642 (5)	0.0399 (4)	0.0263 (10)	
C8	1.2702 (6)	0.5110 (5)	0.1032 (5)	0.0398 (13)	
H8	1.3328	0.4632	0.1511	0.048*	
C9	1.2735 (6)	0.4839 (5)	0.0001 (4)	0.0375 (13)	
H9	1.3389	0.4132	−0.0394	0.045*	
C10	1.1113 (6)	0.6846 (5)	0.2325 (4)	0.0369 (12)	
H10A	1.2010	0.6708	0.2631	0.044*	
H10B	1.0781	0.7800	0.2135	0.044*	
C11	0.9870 (8)	0.6337 (8)	0.3212 (6)	0.074 (2)	
H11A	0.9042	0.6342	0.2878	0.111*	
H11B	0.9471	0.6884	0.3843	0.111*	
H11C	1.0255	0.5439	0.3516	0.111*	
C12	1.1316 (6)	0.5854 (5)	−0.1481 (4)	0.0313 (11)	
H12A	1.0230	0.6247	−0.1405	0.047*	
H12B	1.1532	0.4971	−0.1697	0.047*	
H12C	1.1970	0.6390	−0.2081	0.047*	

### Atomic displacement parameters (Å^2^)

**Table:**

	*U*^11^	*U*^22^	*U*^33^	*U*^12^	*U*^13^	*U*^23^
Ag	0.0342 (2)	0.0232 (2)	0.0293 (2)	0.00745 (15)	−0.01754 (17)	−0.00302 (15)
Cl1	0.0483 (7)	0.0219 (6)	0.0319 (7)	0.0102 (5)	−0.0150 (6)	−0.0023 (5)
Cl2	0.0273 (6)	0.0564 (9)	0.0366 (7)	−0.0102 (6)	−0.0147 (5)	0.0073 (6)
Cl3	0.0295 (6)	0.0343 (7)	0.0365 (7)	−0.0071 (5)	−0.0155 (5)	−0.0084 (5)
P1	0.0282 (6)	0.0178 (6)	0.0176 (6)	0.0025 (5)	−0.0096 (5)	0.0003 (4)
P2	0.0234 (6)	0.0208 (6)	0.0185 (6)	0.0026 (5)	−0.0095 (5)	0.0015 (5)
P3	0.0223 (6)	0.0175 (6)	0.0200 (6)	−0.0007 (4)	−0.0114 (5)	0.0005 (4)
O1	0.0401 (19)	0.0250 (17)	0.0163 (17)	0.0015 (14)	−0.0058 (14)	0.0028 (13)
O2	0.0313 (17)	0.0224 (16)	0.0219 (17)	0.0008 (13)	−0.0180 (14)	0.0010 (13)
N1	0.037 (2)	0.025 (2)	0.039 (3)	0.0035 (18)	−0.015 (2)	−0.0027 (19)
N2	0.036 (2)	0.025 (2)	0.035 (2)	−0.0018 (18)	−0.0168 (19)	0.0090 (18)
N3	0.030 (2)	0.033 (2)	0.027 (2)	0.0016 (17)	−0.0141 (18)	−0.0029 (18)
N4	0.029 (2)	0.027 (2)	0.022 (2)	0.0010 (17)	−0.0080 (17)	0.0022 (16)
N5	0.0208 (18)	0.022 (2)	0.022 (2)	0.0016 (15)	−0.0099 (15)	−0.0025 (15)
N6	0.032 (2)	0.024 (2)	0.0180 (19)	0.0097 (16)	−0.0143 (16)	0.0007 (15)
N7	0.0247 (19)	0.0208 (19)	0.0169 (19)	−0.0001 (15)	−0.0103 (15)	−0.0007 (15)
C1	0.034 (3)	0.023 (2)	0.033 (3)	−0.003 (2)	−0.019 (2)	0.000 (2)
C2	0.039 (3)	0.027 (3)	0.058 (4)	0.005 (2)	−0.010 (3)	0.002 (3)
C3	0.033 (3)	0.032 (3)	0.048 (4)	0.000 (2)	−0.015 (3)	0.018 (3)
C4	0.053 (4)	0.041 (3)	0.045 (3)	−0.003 (3)	−0.015 (3)	−0.018 (3)
C5	0.088 (5)	0.062 (4)	0.055 (4)	−0.026 (4)	−0.032 (4)	−0.004 (3)
C6	0.076 (4)	0.048 (4)	0.033 (3)	−0.007 (3)	−0.030 (3)	0.006 (3)
C7	0.025 (2)	0.027 (3)	0.025 (2)	−0.0039 (19)	−0.010 (2)	0.0021 (19)
C8	0.034 (3)	0.040 (3)	0.044 (3)	0.010 (2)	−0.024 (3)	−0.001 (3)
C9	0.035 (3)	0.036 (3)	0.031 (3)	0.013 (2)	−0.011 (2)	−0.003 (2)
C10	0.037 (3)	0.045 (3)	0.031 (3)	−0.002 (2)	−0.017 (2)	−0.008 (2)
C11	0.086 (5)	0.110 (6)	0.034 (4)	−0.055 (5)	0.013 (3)	−0.029 (4)
C12	0.032 (3)	0.040 (3)	0.016 (2)	−0.004 (2)	−0.003 (2)	0.000 (2)

### Geometric parameters (Å, °)

**Table:**

Ag—C1	2.065 (5)	N5—P3^i^	1.554 (4)
Ag—C7	2.070 (5)	N7—P3^i^	1.594 (4)
Cl1—P2	2.0118 (16)	C2—C3	1.331 (8)
Cl2—P2	2.0171 (17)	C2—H2	0.9500
Cl3—P3	2.0217 (16)	C3—H3	0.9500
P1—O1	1.468 (3)	C4—C5	1.432 (9)
P1—N5	1.610 (4)	C4—H4A	0.9900
P1—N6	1.615 (4)	C4—H4B	0.9900
P1—O2	1.657 (3)	C5—H5A	0.9800
P2—N6	1.555 (4)	C5—H5B	0.9800
P2—N7	1.578 (4)	C5—H5C	0.9800
P3—N5^i^	1.554 (4)	C6—H6A	0.9800
P3—O2	1.578 (3)	C6—H6B	0.9800
P3—N7^i^	1.594 (4)	C6—H6C	0.9800
N1—C1	1.341 (6)	C8—C9	1.338 (8)
N1—C2	1.365 (6)	C8—H8	0.9500
N1—C4	1.454 (7)	C9—H9	0.9500
N2—C1	1.348 (6)	C10—C11	1.448 (8)
N2—C3	1.374 (6)	C10—H10A	0.9900
N2—C6	1.475 (7)	C10—H10B	0.9900
N3—C7	1.345 (6)	C11—H11A	0.9800
N3—C8	1.385 (6)	C11—H11B	0.9800
N3—C10	1.465 (6)	C11—H11C	0.9800
N4—C7	1.347 (6)	C12—H12A	0.9800
N4—C9	1.374 (6)	C12—H12B	0.9800
N4—C12	1.464 (6)	C12—H12C	0.9800
			
C1—Ag—C7	178.72 (19)	C5—C4—H4A	109.4
O1—P1—N5	116.2 (2)	N1—C4—H4A	109.4
O1—P1—N6	113.33 (19)	C5—C4—H4B	109.4
N5—P1—N6	112.96 (19)	N1—C4—H4B	109.4
O1—P1—O2	104.90 (18)	H4A—C4—H4B	108.0
N5—P1—O2	104.43 (17)	C4—C5—H5A	109.5
N6—P1—O2	103.29 (19)	C4—C5—H5B	109.5
N6—P2—N7	120.75 (19)	H5A—C5—H5B	109.5
N6—P2—Cl1	108.88 (15)	C4—C5—H5C	109.5
N7—P2—Cl1	108.31 (15)	H5A—C5—H5C	109.5
N6—P2—Cl2	110.61 (17)	H5B—C5—H5C	109.5
N7—P2—Cl2	107.54 (15)	N2—C6—H6A	109.5
Cl1—P2—Cl2	98.43 (7)	N2—C6—H6B	109.5
N5^i^—P3—O2	113.38 (18)	H6A—C6—H6B	109.5
N5^i^—P3—N7^i^	119.10 (19)	N2—C6—H6C	109.5
O2—P3—N7^i^	109.28 (18)	H6A—C6—H6C	109.5
N5^i^—P3—Cl3	109.53 (15)	H6B—C6—H6C	109.5
O2—P3—Cl3	97.59 (12)	N3—C7—N4	104.7 (4)
N7^i^—P3—Cl3	105.49 (14)	N3—C7—Ag	127.0 (3)
P3—O2—P1	126.57 (19)	N4—C7—Ag	128.2 (3)
C1—N1—C2	111.3 (5)	C9—C8—N3	106.8 (4)
C1—N1—C4	123.2 (4)	C9—C8—H8	126.6
C2—N1—C4	125.5 (5)	N3—C8—H8	126.6
C1—N2—C3	111.5 (4)	C8—C9—N4	106.6 (4)
C1—N2—C6	124.2 (4)	C8—C9—H9	126.7
C3—N2—C6	124.3 (4)	N4—C9—H9	126.7
C7—N3—C8	110.6 (4)	C11—C10—N3	112.4 (5)
C7—N3—C10	123.6 (4)	C11—C10—H10A	109.1
C8—N3—C10	125.7 (4)	N3—C10—H10A	109.1
C7—N4—C9	111.2 (4)	C11—C10—H10B	109.1
C7—N4—C12	124.6 (4)	N3—C10—H10B	109.1
C9—N4—C12	124.2 (4)	H10A—C10—H10B	107.9
P3^i^—N5—P1	124.3 (2)	C10—C11—H11A	109.5
P2—N6—P1	123.1 (2)	C10—C11—H11B	109.5
P2—N7—P3^i^	118.6 (2)	H11A—C11—H11B	109.5
N1—C1—N2	103.8 (4)	C10—C11—H11C	109.5
N1—C1—Ag	127.0 (4)	H11A—C11—H11C	109.5
N2—C1—Ag	129.2 (4)	H11B—C11—H11C	109.5
C3—C2—N1	107.4 (5)	N4—C12—H12A	109.5
C3—C2—H2	126.3	N4—C12—H12B	109.5
N1—C2—H2	126.3	H12A—C12—H12B	109.5
C2—C3—N2	105.9 (4)	N4—C12—H12C	109.5
C2—C3—H3	127.0	H12A—C12—H12C	109.5
N2—C3—H3	127.0	H12B—C12—H12C	109.5
C5—C4—N1	111.3 (5)		
			
N5^i^—P3—O2—P1	47.2 (3)	C7—Ag—C1—N1	−47 (9)
N7^i^—P3—O2—P1	−88.3 (3)	C7—Ag—C1—N2	133 (8)
Cl3—P3—O2—P1	162.3 (2)	C1—N1—C2—C3	−0.3 (7)
O1—P1—O2—P3	136.3 (2)	C4—N1—C2—C3	−178.1 (5)
N5—P1—O2—P3	13.5 (3)	N1—C2—C3—N2	−0.7 (6)
N6—P1—O2—P3	−104.8 (3)	C1—N2—C3—C2	1.5 (6)
O1—P1—N5—P3^i^	146.1 (3)	C6—N2—C3—C2	−179.9 (5)
N6—P1—N5—P3^i^	12.6 (4)	C1—N1—C4—C5	−84.8 (7)
O2—P1—N5—P3^i^	−98.9 (3)	C2—N1—C4—C5	92.9 (7)
N7—P2—N6—P1	5.5 (4)	C8—N3—C7—N4	0.0 (6)
Cl1—P2—N6—P1	131.7 (2)	C10—N3—C7—N4	−177.7 (4)
Cl2—P2—N6—P1	−121.2 (3)	C8—N3—C7—Ag	−177.6 (4)
O1—P1—N6—P2	−144.9 (3)	C10—N3—C7—Ag	4.7 (7)
N5—P1—N6—P2	−10.1 (4)	C9—N4—C7—N3	−0.2 (6)
O2—P1—N6—P2	102.2 (3)	C12—N4—C7—N3	−179.0 (4)
N6—P2—N7—P3^i^	−2.2 (4)	C9—N4—C7—Ag	177.5 (4)
Cl1—P2—N7—P3^i^	−128.6 (2)	C12—N4—C7—Ag	−1.4 (7)
Cl2—P2—N7—P3^i^	125.9 (2)	C1—Ag—C7—N3	40 (9)
C2—N1—C1—N2	1.2 (6)	C1—Ag—C7—N4	−137 (8)
C4—N1—C1—N2	179.1 (5)	C7—N3—C8—C9	0.1 (6)
C2—N1—C1—Ag	−178.8 (4)	C10—N3—C8—C9	177.7 (5)
C4—N1—C1—Ag	−0.9 (7)	N3—C8—C9—N4	−0.2 (6)
C3—N2—C1—N1	−1.6 (6)	C7—N4—C9—C8	0.2 (6)
C6—N2—C1—N1	179.8 (5)	C12—N4—C9—C8	179.0 (5)
C3—N2—C1—Ag	178.3 (4)	C7—N3—C10—C11	87.5 (7)
C6—N2—C1—Ag	−0.2 (8)	C8—N3—C10—C11	−89.9 (7)

Symmetry codes: (i) −*x*+1, −*y*+1, −*z*+1.
